# Are Gyms a Feasible Setting for Exercise Training Interventions in Patients with Cardiovascular Risk Factors? An Italian 10-Years Cross-Sectional Survey Comparison

**DOI:** 10.3390/ijerph19042407

**Published:** 2022-02-19

**Authors:** Marco Vecchiato, Giulia Quinto, Stefano Palermi, Giulia Foccardi, Barbara Mazzucato, Francesca Battista, Federica Duregon, Federica Michieletto, Daniel Neunhaeuserer, Andrea Ermolao

**Affiliations:** 1 Sports and Exercise Medicine Division, Department of Medicine, University of Padova, Via Giustiniani 2, 35128 Padova, Italy; marcovecchiato.md@gmail.com (M.V.); giulia.quinto9@gmail.com (G.Q.); giulia.foccardi@gmail.com (G.F.); barbara.mazzucato.9178@gmail.com (B.M.); francescabatt85@yahoo.it (F.B.); federica.duregon@unipd.it (F.D.); andrea.ermolao@unipd.it (A.E.); 2 Clinical Network of Sport and Exercise Medicine of the Veneto Region, 35131 Padova, Italy; 3 Public Health Department, University of Naples Federico II, 80131 Naples, Italy; stefanopalermi8@gmail.com; 4 Directorate of Prevention, Food Safety, and Veterinary Public Health, Veneto Region, 35123 Venice, Italy; federica.michieletto@regione.veneto.it

**Keywords:** CV risk factor, fitness center, prevention, physical activity, exercise

## Abstract

Background: Exercise training is a known important prevention and treatment modality in patients with cardiovascular (CV) diseases. However, the CV risk factors in gym users have been poorly studied. The aim of this study was to monitor CV risk factors of gym users over 10 years in order to investigate whether gyms are used settings for secondary disease prevention. Methods: In 2007 and 2017, a cross-sectional research survey was adopted to determine CV risk factors and habits in gym users (18–69 years) of the Veneto region. These data were analyzed and compared with those of PASSI, a national surveillance system of the Italian population. Results: During the last decade, there has been an increase in gym users over 50 years of age and in people with arterial hypertension and hypercholesterolemia. People attending the gym on medical referral are increasing, but they are still few (<10%). When comparing the collected data with PASSI surveillance, most of the CV risk factors are strongly underrepresented in gym users. Conclusion: The prevalence of gym users with CV risk factors is rather low, regardless of age. Physicians still need to encourage and prescribe physical exercise for secondary prevention and treatment of chronic diseases.

## 1. Introduction

Physical activity (PA) and exercise training play a central role in health maintenance as well as in primary and secondary disease prevention. Indeed, current first-line treatments of arterial hypertension, hypercholesterolemia, obesity, diabetes, and nonalcoholic fatty liver disease rely on dietary management, lifestyle modification and, particularly, physical exercise [1,2]. Several physiological mechanisms are involved, and the impact of PA to decrease cardiovascular (CV) risk factors has been largely demonstrated, and also for different exercise modalities [3,4]. Gyms and fitness centers are now widely accessible in big cities and many small towns and play a critical role in implementing strategies to reduce the population’s CV risk factors [5,6]. Moreover, these places have considerably changed during the last two decades: from being spaces for fitness lovers, designed for a target audience of young male customers, they advanced into a broader concept and range of products regarding exercise that now attracts all age groups and increasingly appeals to the female gender [7]. In a recent European survey, health-oriented fitness trends appear very attractive, demonstrating that more than one-third of the top trends are associated with exercise for health issues [8]. Thus, although the subset of gym users today might represent the general active population, these facilities could also represent settings for secondary prevention since exercise should be considered as a cost-effective medical intervention that could be prescribed as a first-line treatment for almost every chronic disease [9]. Indeed, subjects with CV risk factors or even patients with non-communicable diseases (NCDs) should also attend gyms, which might thereby support the implementation of exercise as medicine. However, to the best of our knowledge, the characterization of subjects frequenting gyms to evaluate the prevalence of CV risk factors, NCDs or other previous diseases has never been reported in the literature.

NCDs are one of the toughest challenges in public health throughout the world as well as in Europe and in Italy [10,11,12,13], and many current guidelines underline the cardinal role of PA and exercise in primary prevention, rehabilitation and secondary prevention [14,15]. From 2005, the World Health Organization (WHO) officially adopted the term “public health surveillance” to encourage many countries to activate dynamic behavioral surveillance, monitoring the modifiable NCDs risk factors [16,17]. Currently, the vast literature shows the crucial importance of these surveillance systems in public health [18]. Through continuous data collection, surveillance systems are intimately linked to public health issues, as they provide relevant information regarding health promotion and facilitate decision-making processes.

PASSI (Progresses in ASSessing adult population health in Italy) is a surveillance system funded in 2007 by the Ministry of Health and coordinated by the Italian National Institute of Health [19]. It investigates various topics, including the PA level and CV risk factors. In January 2018, PASSI celebrated ten years of activity, with the number of participants consistently growing over time [20]. Therefore, PASSI surveillance appears to be a useful tool for analyzing population habits and changes during time, also related to CV disease prevention.

The aim of the present study was to monitor the characteristics of gym users over 10 years, trying to understand if subjects with CV risk factors or chronic diseases attend fitness centers in order to maintain their recommended PA level and prevent develop of NCDs.

## 2. Materials and Methods

This is a long-term observational study organized by the Sports and Exercise Medicine Division of the University of Padova. Two cross-sectional survey evaluations were performed in 2007 and 2017 in order to investigate characteristics, CV risk factors and habits among gym users in the Veneto region. Secondly, both surveys were separately compared with data of the general population obtained from the PASSI surveillance system, also considering territory, age and year of investigation.

### 2.1. Survey

For the aims of this study, a specific survey was designed and edited in the participants’ native language (Italian). The survey was anonymous, and participation was on a voluntary basis. The survey was structured into 10 items, recording gender, age, presence of six CV risk factors (arterial hypertension, hypercholesterolemia, overweight/obesity, diabetes, smoking habits, heart diseases), gym membership reasons (i.e., weight loss, aesthetic purposes, entertainment/fun, social relations, medical referral, other (to specify—multiple choices were possible)) and the evidence of benefits from PA (Yes/No). Arterial hypertension was defined as systolic blood pressure values > 140 mmHg and/or diastolic blood pressure values > 90 mmHg, also considering those subjects receiving antihypertensive therapy [21]. Patients were registered as hypercholesterolemic when low-density lipoprotein cholesterol values were > 116 mg/mL or receiving lipid-lowering therapy [22]. Overweight was defined as a body mass index greater than 25 kg/m^2^ [23]. Patients with a fasting plasma glucose ≥ 7.0 mmol/L (126 mg/dL) and those receiving hypoglycemic therapy were registered as diabetics [24]. Subjects were defined as smokers with a smoking history of at least 100 cigarettes (5 packs) in their lifetime and/or when being a smoker at the time of the interview; subjects who had quit smoking in the last 6 months from the assessment were registered as smokers too [25]. Heart diseases were defined as a range of conditions, including coronary artery disease, heart failure, arrhythmias, congenital heart defects, heart valve diseases or related infections [26]. The definitions of these clinical conditions were intentionally simplified to make them comprehensible to all respondents and were kept unchanged between the two surveys.

This survey was administered in 11 gyms in Padova and Venice districts for a period of one year. While in 2007, a paper format was used for the survey; in 2017, an online model was additionally available for the participants via the tool “SurveyMonkey” [27]. Data were only evaluated and elaborated when participants answered all questions.

### 2.2. PASSI Surveillance

The PASSI project is characterized by public health surveillance that collects, continuously and through sample surveys edited by telephone interviews, information from the Italian adult population on lifestyles and behavioral risk factors related to the onset of chronic NCDs, as well as preventive strategies and access to health care. PASSI collects data from representative samples of non-institutionalized adult citizens aged 18–69 years, also considering age groups, gender and region of origin. National data of PASSI surveillance were free, public and archived as annual summary national reports until 2013, while thereafter national data can be consulted at the PASSI online web page [28].

### 2.3. Statistical Analysis

Data collected by the two surveys were extracted in Microsoft Excel data sheets. Continuous variables were summarized and reported as mean and standard deviation. Frequencies were reported as percentages. Categorical variables were compared between groups using Pearson’s Chi-squared test. Statistical analyses were performed with the Statistical Package for Social Science (SPSS Inc., Chicago, IL, USA ver. 20). All reported probability values are two-tailed, and a value of *p* < 0.05 was considered statistically significant.

## 3. Results

### 3.1. What Has Changed in the Last Decade?

In 2007, the survey was completed by 1049 participants: 472 females (45%), with a mean age of 37.2 (range 18–69) years. In 2017 the survey was completed by 919 participants: 441 females (48%), with a mean age of 43.9 (range 19–67) years. Overall data concerning the results of both surveys are shown in Table 1.

The percentage of gym users over 50 years of age increased significantly in the last decade (9.9% vs. 21.0%; *p* < 0.001). The same trend was reported for subjects going to gyms on medical referral (5.2% vs. 7.7%; *p* = 0.035). Gym users affected by arterial hypertension increased significantly in the last 10 years (4.9% vs. 8.6%; *p* = 0.001), as well as gym users with hypercholesterolemia (5.9% vs. 11.8%; *p* < 0.001). No differences were observed regarding other comorbidities, such as diabetes and overall heart diseases, as well as in the smoking habits of this population.

### 3.2. Survey vs. PASSI Surveillance

The comparison between the gym population observed through our surveys and the data of the general population extracted from PASSI surveillance for the Veneto Region is shown in Table 2.

**Figure 1:**
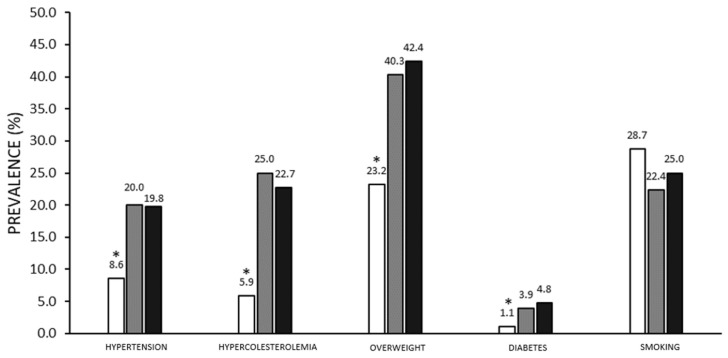
Cardiovascular risk factors in gym users and the general population. Comparison between CV risk factors investigated in the 2017 Survey (white columns) on gym users and 2017 PASSI surveillance data for the Veneto region (grey columns) and Italy (black columns). * *p* < 0.05 between Survey and PASSI surveillance data (Veneto region and Italy).

Subjects’ characteristics seem to be different regarding the prevalence of arterial hypertension, hypercholesterolemia and overweight. No significant differences emerged for smoking habits. Focusing on the most recent data of our survey and comparing these characteristics with Veneto Region and the Italian population drawn from PASSI surveillance, all CV risk factors, except smoking, are significantly under-reported in gym users (Figure 1). No gender differences can be observed in the three groups.

## 4. Discussion

The present study shows some small but promising changes in gym users during the last decade. Moreover, although patients with CV risk factors are still strongly underrepresented in gyms compared to the general population of the same investigated territory, these settings are slowly becoming more attractive for secondary disease prevention.

When observing data between 2007 and 2017, gender distribution seems to remain stable over time, while the mean age of gym users changed. In particular, it is interesting to note an increase in the proportion of gym users over 50 years of age. This change entails an unavoidable surge in CV risk factors among the population studied, such as arterial hypertension and hypercholesterolemia. The age differences between surveys could be partially explained by an increase in average age living and thus an ever-rising demand for active living and health of this age classes. In recent years, gyms and fitness centers responded to this new request and offered more services and adapted settings for all age groups [29]. No differences in people with diabetes and heart diseases emerged within the last decade, and considering the importance of PA in these pathologies and the number of patients in the general population, the absolute prevalence of these diseases in gyms should be much higher. One reason could be ascribed to the fear of patients when performing unsupervised exercise in a place historically associated with recreationally active or experienced exercisers as well as athletes. Indeed, there are still too many people who currently underestimate the positive role of PA against several diseases [30]. However, the evidence of the beneficial effects of regular PA in the prevention and treatment of most diseases and in subjects’ psychophysical well-being is strong and reflected in our results, which show a 10-years increase in the percentage of people enrolled in gyms after medical referral. Data revealed that these suggestions were only expressed verbally with no written exercise prescription and without specific recommendations regarding aerobic or resistance training. However, this may represent an encouraging sign that physicians can play a key role in stimulating patients to PA, and gyms could be better exploited for implementation strategies in secondary disease prevention.

PASSI surveillance divides the population into three categories: physically active, partially active and sedentary. “Physically active” people are those respondents who declare practicing PA according to the WHO recommendations, or they carry out a work activity that requires significant physical effort. People defined as “partially active” are those who do not perform heavy work but engage in leisure-time PA, not reaching recommended levels. People classified as “sedentary” do not engage in any leisure-time PA, nor perform heavy work. Gym users investigated in our surveys could be classified as physically active or partially active, as they represent only a subgroup of what was reported in 2017 PASSI surveillance. This could represent a partial selection bias because it is reasonable to assume that the sedentary population, lacking in our sample, presents a higher prevalence of CV risk factors. Sub-classifying gym users by age groups may partially reduce the aforementioned selection bias. However, the differences in CV risk factor prevalence between gym users and the general population remain significant for all age groups and are particularly remarkable for subjects over 50 years of age. The reasons for this under-representation of CV risk factors may be manifold, but two main hypotheses can be proposed.

The first assumption is that gym users represent an active and health-oriented subset of the general population leading to beneficial adaptations on CV risk factors. Even when compared in terms of age and gender, gyms are frequented by a selected active population, which therefore cares more about health issues, treats risk factors more carefully and generally recognizes a preventive and therapeutic role of PA and exercise [31,32]. The intrinsic role of regularly performed PA that has a strong impact on reducing all CV risk factors should be added to this fact. In fact, there is a low prevalence of overweight subjects (25% in gyms versus 40% in the general PASSI population), which has remained unchanged over the years, being particularly evident in the over-50s group.

The second scenario is that fewer people with CV risk factors attend the gyms. This option can be considered realistic as all NCDs and CV risk factors as arterial hypertension, hypercholesterolemia, overweight and diabetes, are significantly underrepresented, independently of age. On the other hand, smoking habits have remained similar throughout the years and were comparable with those of the general population. However, despite the benefits of supervised exercise compared to self-administered exercise being proven [33,34,35], the gym setting might appear unfriendly and does not provide enough incentives for patient participation, and the promotion of exercise as a health benefit seems also limited [36]. Moreover, it is important to consider that personal trainers generally do not possess the same theoretical and practical background regarding patients with chronic diseases, and many of them do not have a specific academic qualification or specialization. In order to increase the safe enrolment of patients within gyms, the Veneto Region started in 2015 to recognize some specific gyms as “Palestre della Salute” (Health Gyms), if equipped with adequate facilities and kinesiologist trained in the administration of adapted exercise in chronic diseases [37]. In order to connect these Health Gyms with public healthcare, medical doctors are needed to assign their patients to these gyms with a written exercise prescription. According to patients’ risk stratification and professional abilities, the exercise prescription can also be provided by the general practitioner or by other medical specialists. It has to be determined by a further regional follow-up analysis whether this implementation strategy will lead to a promotion of gyms for secondary prevention in patients with CV risk factors or diseases.

Another important aspect to consider is the underestimation of the cardinal role of PA in the prevention and management of NCDs by healthcare professionals [38]. In order to include PA and exercise as a standard in the healthcare system, during the last few years, different initiatives have progressively emerged all over the world [39,40,41,42]. One of the main goals of these initiatives is promoting PA through linking patients from hospitalized settings to local level resources as a continued care model. In order to work on the effective implementation of PA in public healthcare, a collaboration of policymakers, healthcare professionals, exercise specialists and community facilities is needed. However, recent data emphasized how all aspects of exercise prescription are provided heterogeneously in primary care, with a lack of standardization and limited specific knowledge and abilities, which will certainly affect the outcomes of a training intervention and its beneficial effects [38,43]. It is also necessary to find ways to bridge the gap between clinicians and exercise professionals as public health strategies are not yet adequately relying on a partnership with gym clubs to promote PA for patients [44]. Moreover, a good part of the population with CV risk factors seems to be unaware of the key role that exercise training can have for primary and secondary disease prevention and how much this contributes to improving, and in some cases removing, risk factors [30]. Thus, the Health Gyms model, in which a qualified staff receives a written medical exercise prescription for an individualized training adaptation, could be a promising approach to improve patients’ awareness regarding the importance of health-related PA, thereby also increasing the impact on the territory.

### Limitations and Perspectives

This study comes with some limitations due to the characteristics of the study population and setting. Moreover, the sample sizes obtained in the two surveys is appropriate but rather inferior to the PASSI data, which has considerably increased the sample over the years (2007 Survey: *n* = 1049 vs. 2007 PASSI: *n* = 3219; 2017 Survey: *n* = 919 vs. 2017 PASSI: *n* = 20059). Furthermore, the comparison with the nationwide Italian PASSI data must consider that the distribution of some CV risk factors is very different between the southern and northern regions of Italy. Future studies may aim to expand the sample size through an online survey involving all Italian regions and other European countries. This survey should also address the role of exercise professionals regarding the implementation of exercise interventions in gyms for public health issues. Future trials evaluating cost-savings and cost-effectiveness would be useful to further promote gyms as a feasible setting for exercise training interventions in patients with cardiovascular risk factors.

## 5. Conclusions

The prevalence of gym users with CV risk factors was found to increase in this 10-year follow-up analysis. However, patients with NCDs and CV risk factors are still not enrolled enough in a supervised training setting within gyms, which should become high-impact facilities for public health strategies. Future initiatives may aim to increase the knowledge and abilities of healthcare professionals and exercise specialists as well as the general population’s awareness concerning the benefits of regular PA for primary and secondary disease prevention. Finally, surveillance systems and analyses are important tools that may facilitate decision-making for public health purposes, particularly useful to promote strategies for implementing exercise training as a treatment modality in healthcare.

## Figures and Tables

**Table 1:** Cardiovascular risk factors in gym users.

	2007 Survey *n* = 1049	2017 Survey *n* = 919	*p*
Gender (female)	472 (45.0)	441 (48.0)	0.422
Age			
18–35 years	667 (63.6)	513 (55.8)	0.079
35–50 years	273 (26.5)	213 (23.2)	0.187
50–69 years	104 (9.9)	193 (21.0)	<0.001
Arterial hypertension	52 (4.9)	79 (8.6)	0.001
Hypercholesterolemia	62 (5.9)	108 (11.8)	<0.001
Overweight	252 (24.0)	213 (23.2)	0.660
Diabetes	12 (1.1)	13 (1.4)	0.593
Smokers	301 (28.7)	230 (25.0)	0.068
Heart disease	17 (1.6)	13 (1.4)	0.710
Medical referral	55 (5.2)	71 (7.7)	0.035
Benefits from PA	1026 (97.8)	906 (98.6)	0.938

Table 1 shows the 2007 and 2017 Survey data collected in gym users of the Veneto region. Data are expressed as frequencies and percentages. There is a significant increase in gym users over the age of 50, for those with hypercholesterolemia and arterial hypertension, with a consensual increase in subjects attending the gym on medical referral (PA: Physical activity).

**Table 2:** Comparison between gym users and the general population.

	2007 Survey *n* = 1049	2007 PASSI *n* = 3219	*p*	2017 Survey *n* = 919	2017 PASSI *n* = 20059	*p*
Hypertension	52 (4.7)	731 (22.7)	<0.001	79 (8.6)	4012 (20)	<0.001
18–35 years	15 (2.2)	72 (7.4)	<0.001	3 (0.5)	180 (3.5)	<0.001
35–50 years	22 (7.9)	186 (16.5)	<0.001	21 (9.9)	893 (13.1)	0.167
50–69 years	15 (14.4)	473 (42.5)	<0.001	55 (28.5)	2939 (36.3)	0.026
Male	36 (6.2)	388 (23.8)	<0.001	40 (8.4)	1872 (18.9)	<0.001
Female	16 (3.4)	343 (21.6)	<0.001	39 (8.8)	2140 (21.0)	<0.001
Hypercholesterolemia	62 (5.9)	927 (28.8)	<0.001	108 (11.8)	4594 (22.9)	<0.001
18–35 years	24 (3.6)	141 (14.4)	<0.001	22 (4.3)	406 (7.9)	0.003
35–50 years	26 (9.4)	272 (24.1)	<0.001	34 (16.0)	1289 (18.9)	0.280
50–69 years	12 (11.5)	514 (46.2)	<0.001	52 (26.9)	2899 (35.8)	0.011
Male	29 (5.0)	266 (28.6)	<0.001	44 (9.2)	2240 (22.7)	<0.001
Female	33 (7.0)	461 (29.0)	<0.001	64 (14.5)	2354 (11.7)	<0.001
Overweight	252 (24.0)	1352 (42.0)	<0.001	213 (23.2)	8084 (40.3)	<0.001
18–35 years	135 (20.2)	225 (23.0)	0.186	108 (21.1)	1150 (22.4)	0.486
35–50 years	81 (29.1)	431 (38.2)	0.005	71 (33.3)	2612 (38.3)	0.142
50–69 years	36 (34.6)	696 (62.5)	<0.001	34 (17.6)	4322 (53.3)	<0.001
Male	186 (32.2)	828 (50.8)	<0.001	116 (24.3)	4925 (49.9)	<0.001
Female	66 (14.0)	524 (33.0)	<0.001	97 (22.0)	3159 (31.0)	<0.001
Diabetes	12 (1.1)	129 (4.0)	<0.001	13 (1.4)	943 (4.7)	<0.001
18–35 years	4 (0.6)	5 (0.5)	0.058	2 (0.4)	62 (1.2)	0.095
35–50 years	2 (0.7)	32 (2.8)	0.039	1 (0.5)	136 (2.0)	0.113
50–69 years	6 (5.8)	92 (8.3)	0.371	10 (5.2)	745 (9.2)	0.055
Male	7 (1.2)	72 (4.4)	<0.001	6 (1.3)	577 (5.8)	<0.001
Female	5 (1.1)	57 (3.6)	0.009	7 (1.6)	366 (3.6)	0.025
Smokers	301 (28.7)	827 (25.7)	0.055	230 (25.0)	4509 (22.5)	0.071
18–35 years	212 (31.8)	340 (34.7)	0.214	150 (29.2)	1532 (29.8)	0.779
35–50 years	66 (23.7)	299 (26.5)	0.342	44 (20.7)	1555 (22.8)	0.462
50–69 years	23 (22.1)	188 (16.9)	0.178	36 (18.7)	1422 (17.5)	0.690
Male	170 (29.5)	470 (28.9)	0.781	133 (27.8)	2595 (26.3)	0.458
Female	131 (27.8)	357 (22.5)	0.018	97 (22.0)	1914 (18.8)	0.092

Table 2 shows a comparison between the collected data in gym users and those from the general population revealed by the PASSI surveillance in the Veneto region for 2007 and 2017, respectively. The examined CV risk factors were compared by stratifying the samples by age and gender. Data are expressed as frequencies and percentages.

## Data Availability

The data that support the findings of this study are available from the corresponding author upon reasonable request.
